# HPV *E6/E7*, *hTERT*, and *Ki67* mRNA RT-qPCR Assay for Detecting High-Grade Cervical Lesion with Microscope Slides

**DOI:** 10.1155/2019/9365654

**Published:** 2019-01-14

**Authors:** Geehyuk Kim, Jemberu Taye, Kwangmin Yu, Sunyoung Park, Jungho Kim, Sunghyun Kim, Dongsup Lee, Hye-young Wang, Kwang Hwa Park, Hyeyoung Lee

**Affiliations:** ^1^Department of Biomedical Laboratory Science, College of Health Sciences, Yonsei University, Wonju, Gangwon Province, Republic of Korea; ^2^Department of Pathology, Yonsei University Wonju College of Medicine, Wonju, Gangwon Province, Republic of Korea; ^3^Department of Clinical Laboratory Science, College of Health Sciences, Catholic University of Pusan, Busan, Republic of Korea; ^4^Department of Clinical Laboratory Science, Hyejeon College, Hongseong, Chungnam Province, Republic of Korea; ^5^M&D, Inc., Wonju Eco Environmental Technology Center, Wonju, Gangwon Province, Republic of Korea

## Abstract

After breast and colon cancer, cervical cancer is the third most common cancer of women worldwide. Since human papillomavirus (HPV) infection is known to be the predominant cause of cervical cancer, molecular HPV screening is currently used along with cytological and histological examination methods for precancer diagnosis. Nevertheless, the sensitivity of the current HPV test is less than 80%; thus, many cervical cancer cases are not able to be diagnosed by HPV screening alone, and likewise, patients with cervical cancer are often determined to be HPV-negative by the current screening methods. Therefore, human telomerase reverse transcriptase (*hTERT*) and *Ki67* previously identified as cancer markers were attempted. And cervical exfoliated cells of high-grade squamous intraepithelial lesion (HSIL), the most severe precancerous lesion of cancer, were used in the study. However, it takes a long time to collect enough specimens to conduct statistical analysis. Therefore, in the present study, microscope slides, cervical exfoliated cells on glass slides, were attempted. The results of the analysis demonstrated that *hTERT* and *Ki67* expression levels were useful in distinguishing between cancerous and normal specimens, exhibiting a higher sensitivity and specificity than conventional HPV *E6/E7* testing. And the study suggests clinical slide cell samples could be effectively used in the context of retrospective studies to identify novel biomarkers.

## 1. Introduction

Cervical cancer is the third most common cancer of women worldwide, after breast and colon cancer [1]. The World Health Organization (WHO) estimates that approximately 528,000 women are diagnosed with cervical cancer each year and that the disease results in approximately 266,000 deaths annually.

Known risk factors for cervical cancer include human papillomavirus (HPV) infection, promiscuous intercourse, sexually transmitted disease infection, long-term hormonal contraceptive use, and smoking [2]. Of these risk factors, HPV infection is the predominant cause of cervical cancer. Thus, effective HPV screening is essential to facilitate accurate and rapid precancer diagnosis and is currently used along with cytological and histological examination worldwide [3–6].

Current “gold-standard” methods for precancer diagnosis are cytological and histological examinations. For cytological examination, exfoliated cervical cells are collected by swabbing the cervix (as part of a “Pap-smear” test), before being placed onto a slide to be inspected for abnormalities. In case of histological examination, it is diagnosed via a microscopic examination of a stained tissue biopsy [7]. Both of these precancer diagnosis methods are affected by sensitivity of test itself and examiners' subjectivity.

Recently, a molecular method via the identification of HPV nucleic-acid sequences was developed for use in conjunction with standard cytological and histological examination techniques, commonly [8].

Currently, commonly used diagnostic markers include the HPV-related proteins L1, E6, and E7. Of these, L1 is a major viral capsid protein that is produced in the cytoplasm, before being translocated into the nucleus of intermediate and superficial squamous epithelial cells, as previously visualized using immunochemical staining. E6 and E7 are primary HPV oncoproteins with numerous cellular targets including p53, and the retinoblastoma tumor suppression protein (pRB). E6 inhibits p53 to prevent apoptosis, whereas E7 is the primary transforming protein, and inhibits pRB to regulate cell-cycle arrest [9, 10]. In the previous study, we assessed the efficacy of cervical cancer diagnosis via screening for the mRNA expression of commonly used HPV markers *L1*, *E6*, and *E7*, along with the additional cancer markers human telomerase reverse transcriptase (*hTERT*) and *Ki67.* hTERT represents the catalytic subunit of telomerase. Telomeres are highly specialized structures that are located at chromosome ends and are known to be essential for genome stability [11]. In fact, telomere dysfunction and telomerase activation have been previously implicated in human cancer progression [12]. The expression level of *hTERT* is known to be the rate-limiting factor for human telomerase activity, and as such, likely a more sensitive indicator of telomerase function and activity than the expression levels of other telomerase subunits that are constitutively expressed in both normal and cancer cells [13]. On the other hand, Ki67 is a nuclear antigen expressed during all active phases of the cell cycle (i.e., G1, S, G2, and M) except G0, and thus, its expression level can be used to determine the cell proliferation status and to predict tumor development [14].

Screening of these diagnostic markers may also be of use in assessing the progression of cervical cancer past the midstage, as demonstrated by a previously conducted prospective study of their expression in formalin-fixed paraffin-embedded (FFPE) clinical histological samples [15]. Cytological samples actually used in clinical screening test were also conducted. However, severe precancerous lesion samples were not enough to conduct statistical analysis. Especially it takes a long time to collect high-grade squamous intraepithelial lesion (HSIL) samples which are the most severe precancerous lesion of cancer. Therefore, in the present study, microscope slides were attempted as samples. They are sealed with Canada balsam in a vacuum state which induce longer storage period relatively. And they could be collected quickly and easily through documented clinical information.

In the present study, HPV and cancer markers mentioned above were analyzed with 110 HSIL and 50 normal microscope slides.

## 2. Materials and Methods

### 2.1. Clinical Samples

A total of 110 and 50 slides with exfoliated cervical-cell samples mounted with Canada balsam (Merck, Frankfurter, Germany) were retrospectively obtained from patients diagnosed to HSIL and normal, respectively, between 2000 and 2004, from the Department of Pathology, Yonsei University Wonju Severance Christian Hospital, Wonju, Republic of Korea. Strictly speaking, normal means negative for intraepithelial lesions or malignancy (NILM) in this context. To reduce interpretive diagnostic error, we only utilized HSIL specimen confirmed with cervical intraepithelial neoplasia grade 2 or worse (CIN2+).

All subjects provided written informed consent for their participation in the study, which was approved by the Institutional Ethics Committee at Yonsei University Wonju College of Medicine (approval no. CR315052).

### 2.2. Histological and Cytological Diagnosis

Clinical diagnosis was predominantly determined cytologically using the 2001 Bethesda System terminology; however, cases with available tissue biopsies were also histologically reviewed.

### 2.3. Slide Preparation and Total RNA Extraction

Slides with exfoliated cervical cells (microscope slides) were used for total RNA extraction. Slides were initially placed in coplin jars with xylene (Duksan, Ansan, Republic of Korea) for 4 days to remove their cover clips (which were mounted with Canada balsam). They were then dried (5 min) and placed into a six-well culture plate (SPL Life Sciences Co., Pocheon, Republic of Korea). The Isol-RNA Lysis Reagent (1 mL; 5 Prime, Austin, TX) was added onto each slide, and a 1 mL exfoliated cervical-cell sample was then collected from each slide via scraping (twice) with an autoclaved slide glass. Each collected exfoliated cervical-cell sample was transferred to an RNase-free 1.7 mL tube, lysed via vortexing/repeated pipetting, and allowed to incubate in the reagent (room temperature, 5 min). After the addition of 200 *μ*L of chloroform, the tube was shaken vigorously, incubated (room temperature, 3 min), and then centrifuged (12,000 g, 15 min). The resultant aqueous layer was transferred to a new tube and mixed with an equal volume of isopropanol by inverting the tube. The mixture was incubated (25°C, 10 min) and then centrifuged (12,000 g, 10 min) before the resulting supernatant was removed, and 1 mL of 75% ethanol was added to the remaining pellet. After mixing via tube inversion, the mixture was centrifuged (7,500 g, 5 min), and the supernatant subsequently removed. The remaining RNA pellet was dried and eluted in 25 *μ*L of diethylpyrocarbonate- (DEPC-) treated water (Intron Biotechnology, Seoul, Republic of Korea). The purity and concentration of the extracted total RNA was determined by measuring its absorbance at 260 and 280 nm using an Infinite 200 plate reader (Tecan, Salzburg, Austria). The isolated total RNA was finally stored at −70°C until use. Note that all preparation and handling of total RNA were performed in a laminar flow hood, under RNase-free conditions.

### 2.4. cDNA Synthesis

Complementary DNA (cDNA) was synthesized using an M-MLV Reverse Transcriptase kit (Invitrogen, Carlsbad, CA, USA) and random hexamers (Invitrogen), according to the manufacturer's recommendations. Briefly, 10 *μ*L of total RNA was added to a master mix containing 1 *μ*L of 10 mM dNTP mix (containing 10 mM each of dATP, dGTP, dCTP, and dTTP at a neutral pH), 0.25 *μ*g of random hexamers, and 1 *μ*L of DEPC-treated water in PCR tubes. The reaction mixture was incubated (65°C, 5 min) and then quickly chilled on ice. A mixture of 4 *μ*L of 5× First-Strand Buffer, 2 *μ*L of 0.1 M dithiothreitol (DTT), and 1 *μ*L of M-MLV reverse transcriptase (RT) was added to the reaction mixture, and the cDNA synthesis reaction then performed via cycling at 25°C for 10 min, 37°C for 50 min, and 70°C for 15 min.

### 2.5. HPV Genotyping Using PCR-REBA

A REBA HPV-ID® PCR-REBA test (YD Diagnostic, Yongin, Republic of Korea), in which a “nested PCR” method was used to amplify target regions between MY11-MY9 and GP5-GP6 using two primer pairs, was used for HPV genotyping. PCR was performed using a 20 *μ*L reaction mixture (Genetbio, Daejeon, Republic of Korea) consisting of 2× master mix, 1× primer mixture, 3 *μ*L of sample DNA, and sterile deionized water (DW). This mixture was subjected to PCR cycling conditions comprising 94°C for 5 min (predenaturation), followed by 15 cycles of 94°C for 30 s (denaturation) and 55°C for 30 s (annealing), 45 cycles of 94°C for 30 s (denaturation) and 52°C for 30 s (annealing), and a final cycle of 72°C for 7 min (strand synthesis). The amplified biotin-labeled PCR products were then denatured (25°C, 5 min) in denaturation solution, diluted in 970 *μ*L of hybridization solution, applied to the REBA membrane strip in the blotting tray, and hybridized (50°C, 30 min) to the desired probes. The membrane strips were then washed twice with 1 mL of washing solution (50°C, 10 min, with gentle shaking), before being incubated (25°C, 30 min) with a streptavidin-alkaline phosphatase (AP) conjugate (Roche Diagnostics, Mannheim, Germany) diluted (1 : 2 000) in a conjugate diluent solution (CDS). After two final washes with 1 mL CDS (room temperature, 1 min), colorimetric hybridization signals were visualized via incubation with an NBT/BCIP solution (1 : 50 dilution, Roche Diagnostics) for sufficient time to detect the enzymatic conversion of the NBT/BCIP substrate to its colored form. The resulting band patterns were then read and interpreted.

### 2.6. Multiplex Quantitative Reverse-Transcriptase (RT-Q) PCR Assay

HPV *E6/E7*, *hTERT*, and *Ki67* mRNA expression in cervical specimens was assessed via a multiplex RT-qPCR TaqMan assay that was performed using the CervicGen HPV *E6/E7* and *hTERT-Ki67* mRNA RT-qDx assay kits (Optipharm, Osong, Republic of Korea) and the CFX-96 real-time PCR system (Bio-Rad, Hercules, CA, USA) for thermal cycling and fluorescence detection. Real-time PCR amplification of HPV *E6/E7* mRNA was performed in a reaction mix containing 10 *μ*L of 2× Thunderbird probe qPCR mix (Toyobo, Osaka, Japan), 5 *μ*L each of primer and TaqMan probe mixture, and 5 *μ*L of template cDNA. Real-time PCR amplification of *hTERT* and *Ki67* mRNA was performed in a reaction mix containing 10 *μ*L of 2× Thunderbird probe qPCR mix (Toyobo, Osaka, Japan), 3 *μ*L of primer and TaqMan probe mixture, 2 *μ*L of template cDNA, and sufficient DW to produce a final volume of 20 *μ*L. Positive and negative controls were included throughout the procedure, and likewise, no-template controls (containing sterile DW instead of template DNA) were amplified with each PCR. The utilized PCR cycling conditions comprised 95°C for 3 min, followed by 41 cycles of 95°C for 3 s, and 55°C for 30 s. Each mRNA expression level was quantified by determining the “cycle threshold” (CT), which is the number of PCR cycles required for the fluorescence to exceed a value significantly higher than the background fluorescence. To avoid the generation of false negative results due to mRNA degradation, the expression level of glyceraldehyde-3-phosphate dehydrogenase (*GAPDH*) was used as an internal control. The six samples of HSIL group and four samples of normal group did not show *GAPDH* value; therefore, they were excluded from the following experiments. In other words, researcher performed experiments with 104 HSIL and 46 normal samples. Target gene mRNA expression levels relative to *GAPDH* were automatically calculated according to the comparative C_t_ method, using CFX Manager v1.6 (Bio-Rad) or Genex (Bio-Rad) Software. Gene expression was assessed using the comparative C_t_ (ΔΔC_t_) method, in which mRNA expression levels are represented relative to the expression level of the reference gene. *hTERT* and *Ki67* expression levels in analyzed slide samples from patients without HSIL were considered to indicate the “baseline” expression level for each gene.

### 2.7. Statistical Analyses

Statistical analyses were performed using GraphPad Prism software version 5.02 (GraphPad, La Jolla, CA, USA). Student's *t*-test, 95% confidence interval (CI), and ROC curve were used to assess the statistical significance of generated data. Cohen's kappa coefficient which measures agreement between two raters for qualitative items is also used.

## 3. Results

### 3.1. Histological and Cytological Diagnosis of Clinical Specimens

All analyzed slide samples were confirmed to exhibit the precancerous condition, cervical intraepithelial neoplasia (CIN) III. Furthermore, histological and cytological methods were used to confirm a diagnosis of HSIL among the 110 relevant slides. Patients with HSIL (range 10-79 years-of-age) were found to be predominantly aged between 30 and 39 (36/104 patients, 34.62%) or 40 and 49 (35/110 patients, 33.65%) years. A lesser number of HSIL patients were aged between 20 and 29 (11/104 patients, 10.58%) or 50 and 59 (12/104 patients, 11.54%) years, and very few were aged less than 20 or greater than 59 years (Table 1).

### 3.2. REBA Analysis of the HPV Infection Status of Analyzed HSIL Clinical Specimens

Of the 104 slides with exfoliated HSIL cervical-cell samples, 83 (79.81%) were found to be infected with at least a single HPV genotype, including 26 (25%) that were infected with multiple (i.e., more than two) HPV genotypes. Among these 83 cases, 56 (53.85%) were determined to be infected with a high-risk (HR) HPV genotype, while a single case (0.96%) was shown to be infected with a low-risk (LR) HPV genotype (Table 2). Among the 26 cases found to be infected with multiple HPV genotypes, 21 (20.19%) were shown to be infected with HR-HPV genotypes, five (4.81%) were determined to be infected with both HR- and LR-HPV genotypes, and no cases were found to be infected with LR-HPV genotypes only.

### 3.3. HPV Genotype Distribution in the Analyzed Clinical Samples

**Figure 1:**
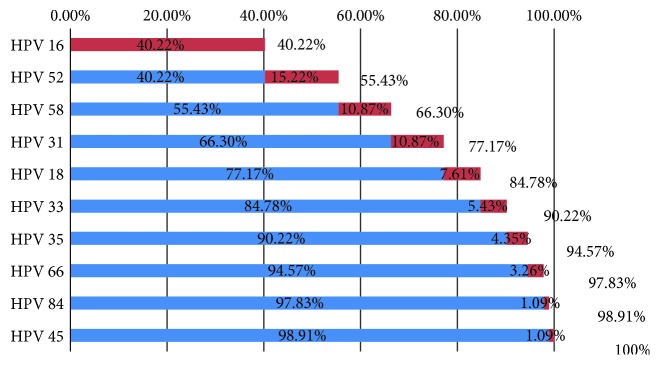
Cumulative graph of human papillomavirus (HPV) genotype distribution among the analyzed high-grade squamous intraepithelial lesion (HSIL) clinical specimens. To inspect HPV distribution, HPV-positive specimens were analyzed in descending order. It is shown as a cumulative graph to check the degree of occupation.

As shown in the constructed cumulative graph (Figure 1), the most frequently detected HPV genotype in the HPV-positive exfoliated cervical-cell samples was HPV *16* (cumulative proportion 40.22%), followed by HPV *52* (55.43%), *58* (66.30%), *31* (77.17%), *18* (84.78%), *33* (90.22%), *35* (94.57%), *66* (97.83%), *84* (98.91%), and *45* (100%).

### 3.4. *hTERT* and *Ki67* mRNA Expression Levels as Determined by RT-qPCR

**Figure 2:**
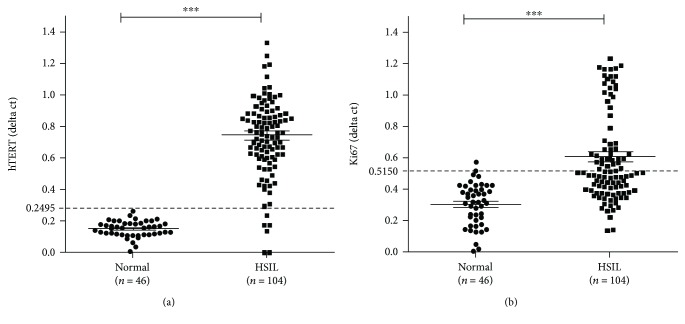
Analysis of the relative expression of *hTERT* (a) and *Ki67* (b) in high-grade squamous intraepithelial lesion (HSIL) versus normal clinical samples using the delta C_t_ method. As shown in (a) and (b), normal and HSIL groups were distinguished by *hTERT* and *Ki67* gene expression showing a statistically significant difference (*p* < 0.001), respectively. The cut-off value for distinguishing between positive and negative results is determined from the receiver operating characteristic (ROC) curve.

We conducted RT-qPCR analyses of *hTERT* and *Ki67* expression levels in the 104 HSIL-diagnosed exfoliated cervical-cell samples. After excluding samples with a no *GAPDH* expression level, we determined that 98 (94.23%) and 49 (47.12%) of the 104 remaining HSIL samples were positive for *hTERT* and *Ki67* mRNA expression, respectively (Figure 2).

### 3.5. Coincident Expression of *hTERT* or *Ki67* with HPV *E6/E7* mRNA in the Analyzed HSIL Clinical Samples

The results of the conducted RT-qPCR analyses showed that of the 104 (nonexcluded) HSIL samples, 80 (76.92%) were positive for both HPV *E6/E7* and *hTERT*, three (2.88%) were positive for HPV *E6/E7* only, 18 (17.31%) were positive for *hTERT* only, and three (2.88%) were positive for HPV *E6/E7*, but negative for *hTERT* (Table 3). Conversely, 38 (36.54%) of the 104 samples were shown to be positive for both HPV *E6/E7* and *Ki67* expression, 45 (43.27%) were positive for HPV *E6/E7* expression only, 11 (10.58%) were positive for *Ki67* expression only, and ten (9.62%) were positive for HPV *E6/E7*, but negative for *hTERT* expression (Table 4).

### 3.6. Combination of HPV *E6/E7*, *hTERT*, and *Ki67* mRNA Expression

**Figure 3:**
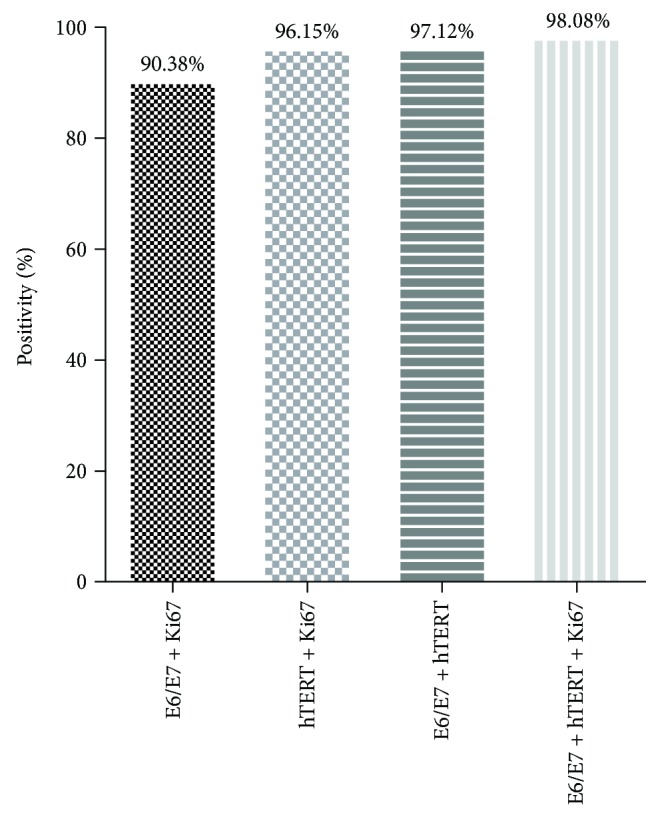
Combined expression patterns of HPV *E6/E7*, *hTERT*, and *Ki67* mRNA in high-grade squamous intraepithelial lesion (HSIL) clinical samples. All of HSIL group could not identify with one marker; therefore, combinational detection of multiple target was tried. Combination of HPV *E6/E7*, *hTERT*, and *Ki67* showed 98.08% (102/104) positive.

The positivity rates were 90.38% (94/104) for a combination of *HPV E6/E7* and *Ki67* mRNA expressions, 96.15% (100/104) for *hTERT* and *Ki67* mRNA expression, 97.12 (101/104) for *HPV E6/E7* and *hTERT* mRNA expressions, and 98.08% (102/104) for the HPV *E6/E7*, *hTERT*, and *Ki67* mRNA expressions (Figure 3).

## 4. Discussion

The previous study was conducted with FFPE clinical tissue samples [16]. It was appropriate to determine the availability of the biomarker by tissue samples because tissue samples collected precisely only cancerous region through microscopy and IHC test. We also confirmed that hTERT and Ki67 mRNA expression could be complementary biomarkers in diagnosing cervical lesions with histological samples. In the present study, microscope slides with exfoliated cervical cell samples were collected from subjects diagnosed as either healthy or with HSIL and screened for HPV genotypes, assessing the usefulness of *hTERT* and *Ki67* expression as diagnostic markers of cervical cancer. Exfoliated cervical cells before being placed onto a slide are currently used for screening test specimen because they accompanied with less invasive and less labor-intensive procedure than other tests [17, 18]. They are exposed to the air and need refrigeration condition, so their storage period is limited. Above all, precancerous lesion samples are relatively infrequent compared to normal samples, and there is no idea how much time needed to collect enough samples for statistical analysis. In fact, HSIL samples, which are the most severe precancerous lesion, were collected over three years in Korea and China, but there were about 50 samples [16, 19]. In contrast, microscope slides are sealed with Canada balsam in a vacuum state, so their storage period is longer relatively. In fact, there was no difference in the housekeeping gene expression between samples by 5 years. And the slides were always prepared for cytology test as routine screening test. These factors enable to collect large number of HSIL samples over 100 easily and immediately. Thus, the slides are adequate specimen for retrospective cohort study. There were several retrospective studies with the slides; however, they are primarily limited to inspect staining of appearance so far [20–23]. For the first time, molecular tests were performed with microscope slides in this study. To verify nucleic acid degradation and variation, the assays were performed with endogenous control genes during the experiment.

The results showed that HR-HPV infection is more closely associated with cervical cancer progression than LR-HPV both in the context of a single or of multiple HPV infections (Table 1). In addition, the most commonly detected HPV genotype among the HPV-positive HSIL specimens was HPV *16,* and notably, detection rate of HPV *18* of slides is lower than that of histological samples.

Cervical cancer oncogenesis is initiated and mediated via the upregulation of the HPV oncoproteins E6 and E7, such that the overexpression of *E6/E7* mRNA transcripts has been shown to be associated with a significantly increased risk of both precancerous group (CIN) and of cervical cancer [24]. The hypothesis that *E6/E7* expression levels may be specific and effective predictors of cervical cancer risk was supported by the results of the present study, which showed the sensitivity of the utilized *E6/E7* mRNA RT-qPCR assay to 79.81% in the 104 analyzed exfoliated cervical-cell samples. It indicate cervical cancer occurrence could be affected other factors not only HPV. Therefore, *hTERT* and *Ki67* confirmed by tissue samples [16] were also applied. The sensitivity of *hTERT* mRNA RT-qPCR screening of the 104 clinical samples was 94.23%, whereas that of *Ki67* screening was only 47.12%. Interestingly, a previous study demonstrated *hTERT* mRNA expression to be higher in cytological than in tissue samples from high-grade cervical lesions, while conversely, *Ki67* mRNA expression was found to be higher in tissue than cytological samples from the high-grade cervical lesions [20]. The mechanism underlying this observed discrepancy between marker genes expression in cytological versus histological samples remains to be elucidated.

While *hTERT* and *Ki67* mRNA expression was only detected in 94.23% and 47.12% of the analyzed cytology samples, respectively, combined screening for HPV *E6/E7* and *Ki67*, HPV *hTERT* and *Ki67*, and HPV *E6/E7* and *hTERT* mRNA expression identified 90.38%, 96.15%, and 97.12% of samples, respectively (Figure 3). Furthermore, coincident screening for HPV *E6/E7*, *hTERT*, and *Ki67* mRNA expression resulted in an RT-qPCR assay sensitivity of 98.08%, suggesting this as a promising combination of markers for the diagnosis of HSIL.

The present study demonstrates the validity of using the non-HPV markers and of analyzing microscope slides for the first time to identify novel diagnostic precancer and cancer biomarkers. Further study is required to assess the suitability of their use as diagnostic markers for low-grade squamous intraepithelial lesions (LSIL) and/or as indicators of the progression of cervical lesions.

## Figures and Tables

**Table 1:** Cytological diagnosis of clinical specimens with respect to patient age.

Patient age (years)	Cytological diagnosis *N* (%)		
	Normal	HSIL	Total
10-19	1 (2.17)	1 (0.96)	2 (1.33)
20-29	3 (6.52)	11 (10.58)	18 (9.33)
30-39	17 (36.96)	36 (34.62)	55 (35.33)
40-49	14 (30.43)	35 (33.65)	51 (32.67)
50-59	8 (17.39)	12 (11.54)	22 (13.33)
60-69	2 (4.35)	8 (7.69)	10 (6.67)
70-79	1 (2.17)	1 (0.96)	2 (1.33)
Total	46 (100)	104 (100)	150 (100)

HSIL: high-grade squamous intraepithelial lesion.

**Table 2:** Distribution of analyzed HSIL specimens with respect to HPV infection type (as assessed via REBA).

HPV infection type			*N* (%)
HPV-positive HSIL *N* = 83/104 (79.81)	Single HPV infection	HR-HPV	56/104 (53.85)
	*N* = 57/104 (54.81)	LR-HPV	1/104 (0.96)
	Multiple HPV infections *N* = 26/104 (25)	HR-HPV	21/104 (20.19)
		LR-HPV	0/104 (0)
		HR- & LR-HPV infections	5/104 (4.81)
HPV-negative HSIL			21/104 (20.19)

HPV: human papillomavirus; REBA: reverse-blot hybridization assay; HSIL: high-grade squamous intraepithelial lesion; HR-HPV: high-risk HPV; LR-HPV: low-risk HPV.

**Table 3:** RT-qPCR analysis of HPV *E6/E7* and *hTERT* mRNA expression in HSIL versus normal clinical specimens.

Cytological diagnosis	HPV *E6/E7*-positive cases		HPV *E6/E7*-negative cases	
	*hTERT*- status, *N* (%)		*hTERT*- status, *N* (%)	
	Positive	Negative	Positive	Negative
HSIL	80/104 (76.92)	3/104 (2.88)	18/104 (17.31)	3/104 (2.88)
Normal	0/46 (0)	2/46 (4.35)	0/46 (0)	44/46 (95.65)

HPV: human papillomavirus; RT-qPCR: quantitative reverse-transcriptase polymerase chain reaction; HSIL: high-grade squamous intraepithelial lesion.

**Table 4:** RT-qPCR analysis of HPV *E6/E7* and *Ki67* mRNA expression in HSIL versus normal clinical specimens.

Cytological diagnosis	HPV *E6/E7*-positive cases		HPV *E6/E7*-negative cases	
	*Ki67*- status, *N* (%)		*Ki67*- status, *N* (%)	
	Positive	Negative	Positive	Negative
HSIL	38/104 (36.54)	45/104 (43.27)	11/104 (10.58)	10/104 (9.62)
Normal	0/46 (0)	2/46 (4.35)	1/46 (2.17)	43/46 (93.48)

HPV: human papillomavirus; RT-qPCR: quantitative reverse-transcriptase polymerase chain reaction; HSIL: high-grade squamous intraepithelial lesion.

## Data Availability

The datasets generated and analyzed during the current study are not publicly available due to patent application but are available from the corresponding author on reasonable request.
